# General public knowledge, attitudes, and practices about rabies and associated factors in Gomma district of Jimma zone, southwestern Ethiopia

**DOI:** 10.1371/journal.pntd.0012551

**Published:** 2024-10-14

**Authors:** Shimelis Kebede, Gashaw Beyene, Biruk Akalu, Elias AbaJebel, Isayas Asefa Kebede

**Affiliations:** 1 Jimma Zone Agriculture Office, Jimma, Oromia, Ethiopia; 2 Ethiopian Ministry of Agriculture, Addis Ababa, Ethiopia; 3 Harari Region Agriculture Office, Harari, Ethiopia; 4 School of Veterinary Medicine, Ambo University, Guder, Ethiopia; Mizan-Tepi University, ETHIOPIA

## Abstract

**Background:**

Rabies is a disease of warm-blooded animals that affects the central nervous system and is almost invariably fatal once clinical signs develop. It is one of the most neglected tropical diseases in several areas of the world, including Ethiopia. Additionally, the burden of the disease is estimated to be high in Ethiopia, and public awareness contributes to prevention.

**Methods:**

A community-based cross-sectional study design was conducted from June–August 2023 in the Gomma district, Jimma zone, Oromia regional state, Ethiopia, to assess the knowledge, attitudes, and practices of communities towards rabies and associated risk factors using a structured questionnaire survey. A multi-stage sampling technique was used for the selection of the sampling units and a total of 140 participants were interviewed. The questionnaire parts included socio-demographics information, knowledge, attitudes, and practices of the community’s respect for rabies management and control. The data was analyzed by Statistical Package for Social Science (SPSS) Version 20. Descriptive analyses were employed and multivariable logistic regression was used to calculate the association between independent and dependent variables (Knowledge, Attitudes, and Practice scores).

**Results:**

About 134 (95.7%) respondents had heard about rabies. Likewise, 75 (53.6%), 55 (39.3%), and 95 (67.9%) of the respondents had good knowledge, favorable attitudes, and good practices, respectively. Christians were more likely to have higher knowledge scores than Muslims (AOR = 6.876, CI = 1.750–27.016, *p* = 0.006). Respondents who knew someone’s exposure had a higher knowledge score than those who did not (AOR = 6.208, CI = 2.750–14.012, *p* = 0.000). Moreover, Muslims were found to have a more favorable attitudes than Christians (AOR = 5.518, CI = 1.199–25.391, *p* = 0.028). Those who knew someone’s exposure to rabies were found to have a more favorable attitudes than those who did not (AOR = 2.367, CI = 1.157–4.839, *p* = 0.018). Respondents who had favorable attitudes towards rabies were found to have more good practices than unfavorable attitudes (AOR = 3.267, CI = 1.391, 7.730, *p* = 0.005).

**Conclusions:**

The study revealed a gap in knowledge among communities in the study area. Thus, rabies control activity and community awareness should be implemented with stakeholders.

## Author summary

In a comprehensive assessment conducted in the Gomma district of Jimma zone, Ethiopia, the community’s knowledge, attitudes, and practices related to rabies were assessed. While participants generally acknowledged domestic dogs and wild carnivores as the primary source of rabies and were able to identify various preventive measures such as avoiding dog bites and ensuring dog confinement, significant disparities were evident. Despite possessing a reasonable level of knowledge, attitudes, and practices were found to be inconsistent with effective rabies prevention. Notably, a prevalent belief persisted among participants that rabies could be cured through traditional herbal medicine, indicating a misconception regarding the disease’s treatment. Additionally, a troubling trend emerged where animals involved in bite incidents were often killed instead of being quarantined, contrary to established guidelines. Recognizing the urgent need for intervention, the study emphasizes the critical role of enhancing awareness and promoting culturally and gender-sensitive educational programs. These initiatives should aim to rectify misconceptions, improve first aid practices, and foster appropriate healthcare-seeking behaviors among community members and healthcare providers alike. By implementing these multifaceted strategies, communities can work towards achieving a significant reduction in the burden of rabies and safeguarding public health.

## Introduction

Rabies has been known since the beginning of human civilization [1]. It is a Central Nervous System (CNS) disease that is almost invariably fatal once a sign develops [1,2]. The causative agent is rabies virus (RABV), a negative-stranded RNA virus of the *Rhabdoviridae* family, genus *Lyssavirus* [2]. Endemic canine rabies is estimated to be the cause of 59,000 human deaths per year across the globe with 56% estimated to be in Asia, and 44% occurring in Africa [3]. About 98% of human rabies cases occur in developing countries that have large numbers of dogs, many of which are stray [4].

In Ethiopia, rabies has been recognized as a very challenging disease for a long time [5]. Furthermore, the distribution of vaccines to the various regions and the fragmented reports on human and animal rabies cases are strong indicators of the widespread nature of the disease in the country [6]. The magnitude of the problem is greater in big cities due to more stray dogs in cities than in rural areas and associated factors [7–10].

In public health, knowledge, attitude, and practice (KAP) studies have been widely used based on the principle that increasing knowledge will result in changing attitudes and practices to minimize disease burden [11]. In Ethiopia, there is some research on rabies KAP [12–15] and elsewhere [16–19]. Furthermore, nationwide data on rabies is not available to reflect the true scope of the disease [14].

Conversely, the distribution of vaccines to the various regions and the fragmented reports on human and animal rabies cases are strong indicators of the widespread nature of the disease in the country [14]. Alie *et al*. [15] reported that inadequate public awareness impeded the implementation of rabies preventive approaches in South Gondar, Ethiopia. These surveys revealed that most people in the community are familiar with rabies, but there are gaps in knowledge and practice about its prevention and control. Moreover, poor public awareness of rabies is considered one of the bottlenecks for the prevention and control of the disease in Ethiopia. Understanding communities’ perceptions of the cause, mode of transmission, symptoms, treatment, and possible intervention measures of rabies is an important step toward developing strategies aimed at controlling the disease and determining the level of implementation of planned activities in the future [14].

Public awareness contributes to disease prevention and control by bridging the information gap in the community. Likewise, enhancing understanding of the community about rabies disease, first aid procedures after dog bites, increased knowledge of dog behavior, and how to avoid being bitten by dogs are suggested approaches to preventing rabies in humans [20].

Although rabies is primarily a disease of dogs in Ethiopia, particularly in the Jimma zone of the Gomma district, no adequate and up-to-date research has been done to address the knowledge gap on the disease through assessing the knowledge, attitude, and practice of the community toward the disease in the Gomma district. Thus, the objectives of the current study were to assess and identify factors associated with knowledge, attitudes, and practices towards rabies among communities in the Gomma district, Jimma zone of the Oromia regional state, southwestern Ethiopia.

## Methods

### Ethical consideration

The study received oral ethical approval (ERC/17/5/7/23) from Addis Ababa University’s Research and Publication Office. Participants provided oral informed consent after being briefed on a written consent form, a method approved by the ethical committee due to challenges with obtaining written consent. Interviewers signed the forms on behalf of the participants, confirming their consent. The consent form detailed the study’s objectives, confidentiality measures, and participants’ rights to refuse or withdraw. This approach ensured ethical compliance while addressing literacy limitations, fostering a respectful and inclusive research process.

### Study sites

**Fig 1:**
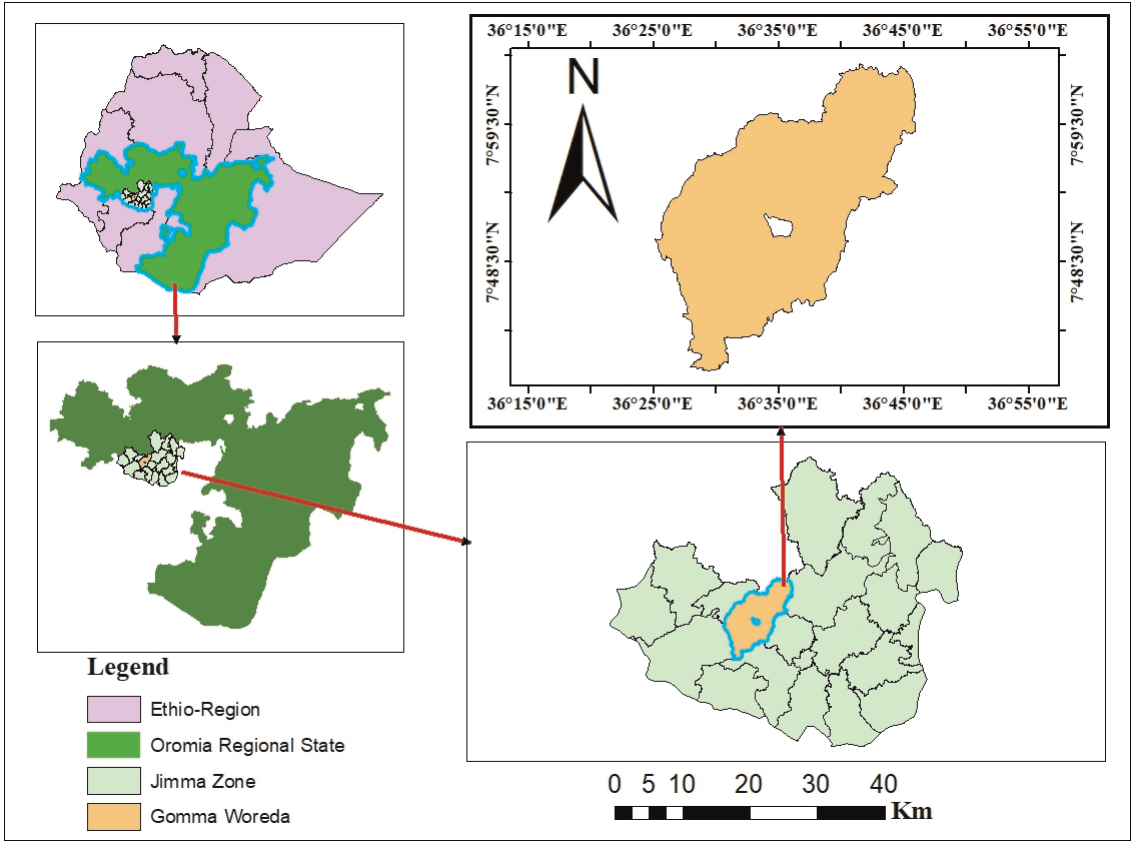
https://data.kimetrica.com/dataset/ethiopia-administrative-units; Fig 1. tif. Inserts showing the study site (Gomma district) within Ethiopia.

The study was conducted in the Gomma district, Jimma zone, Oromia regional state, Ethiopia (Fig 1). The district is located at a distance of 45 km from Jimma town and 400 km from the capital Addis Ababa city and shares a border with Seka Chekorsa, Gera, Setema, Limmu Kosa, and Mana in south, southwest, northwest, northeast, and east directions, respectively. In the northern direction, there is the Didessa River, which separates the district from the Bunno Bedelle zone [21].

The altitude ranges from 1,380 to 2,870 meters above sea level between 07°49’N to 59°99’N latitude and 36°39’ to 59°99’E longitude. The topography includes 4% Kola (lowland), 88% Woinadega (midland), and 8% Dega (highland), with annual rainfall ranging from 1700–2000 mm and temperatures from 13–26°C [21]. A survey of the land in this district shows that 68,750 hectares are arable or cultivable, while 12,418 are considered swampy, mountainous, or otherwise unusable. The majority of the inhabitants are Muslims (83.88%), followed by Christians (14.68%), and Protestants (1.34%) [22]. The district has 36 rural and 5 urban peasant associations (PAs) and a total of 66,094 households with 64,678 and 1,416 male and female households, respectively [21]. The district’s total population is 317,184 of which 161,512 are males and 155,672 are females and 16.3% of its population were urban residents [21]. According to Central Statistical Agency (CSA) of 2017, livestock population data shows 301,536 poultry, 30,998 sheep, 29,873 cattle, 25,678 goats, 9,421 horses, 7,812 mules, 7,261 donkeys, 2,891 dogs and 1,878 cats [23].

### Study duration

The survey was conducted from June 03-August 28, 2023.

### Study design

A cross-sectional study with a community focus was conducted.

### Study population

The study populations were household heads or their spouses and family members (individuals who lived in the selected PAs of the Gomma district for six months, agreed to participate in the study, and ≥18 years).

### Sample size and sampling methods

The required number of populations was calculated using the formula given by Arsham [24].

N = 0.25/SE^2^, Where N = sample size, SE = standard error, 5%. Accordingly, the minimum sample size was 100. However, to increase the precision and representativeness the sample size was increased to 140.

**Fig 2:**
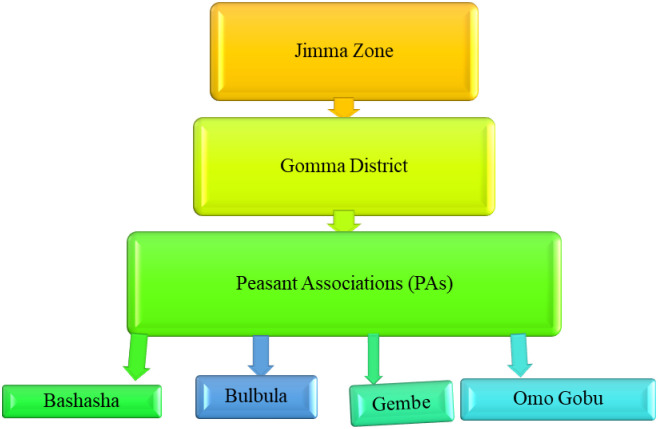
Sampling procedure.

A multi-stage sampling technique was employed for the selection of the sampling units. From the study district, four PAs: namely Bashasha, Gembe, Omo Gobu, and Bulbula were selected through purposive (infrastructures, and densely inhabitants) sampling methods (Fig 2). Then, households in the selected PAs were selected using a systematic random sampling technique. Finally, from all the eligible respondents in a household, only one individual was selected randomly for the interview. Participants were chosen based on their availability at home and eligibility based on age (e.g., ≥ 18 years old with adequate communication and understanding skills). In the absence of eligible respondents in a given household, a replacement was made from the next household until the required sample size was obtained.

### Method of data collection

Data was collected through face-to-face interviews using a pre-tested structured questionnaire. The questionnaire was first prepared in English and translated to Afan Oromo and Amharic for appropriateness and easiness in approaching the study participants. For validation of the questionnaire, a sample of 15 randomly selected individuals in the study area who were not included in the main study were interviewed. The questionnaire was assessed for its understandability, clarity, completeness, reliability, and socio-cultural acceptability. Some questions that were unclear to the respondents were revised. The questionnaire has different sections including socio-demographic factors like residence background, age (18–30 years, 31–45 years, > 45 years), sex (male, female), marital status (single, married, divorced), educational status (unable to read/write, primary school, secondary school, diploma and above), religion (Muslim, Christian), occupation (farmer, daily laborer, student, merchant, civil servant), dog ownership (yes, no), and witness of rabies exposure (yes, no). Moreover, it also incorporated questions concerning the knowledge about rabies etiology, transmission, clinical manifestations, prevention, and control measures both in humans and animals.

To assess the community KAP about rabies each respondent was asked twenty-five questions regarding cause, sources, mode of transmission, clinical signs, prevention practices, and treatment measures. The questions were multiple-choice with close-ended questions. Respondents who answered the questions correctly got one mark and zero for incorrect or do not know responses. Then, the responses for which respondents with correct answers were counted and scored.

The scores were then pooled together and the mean score was computed to determine the overall KAP of respondents. Respondents who scored greater than or equal to the mean value were grouped to good KAP whereas the respondents who scored less than the mean value were grouped to poor KAP level [4,12].

### Data analysis

After collection, the data was cleaned, checked for completeness, and entered into a Microsoft Excel 2016 spreadsheet. The data was then exported and analyzed using Statistical Package for Social Science (SPSS) Version 20. Descriptive statistics was used to calculate the frequency and percentage of both dependent and independent variables. The KAP variables were scored based on respondents’ responses or the number of sources they were familiar with. Yes/No-based assessments of rabies awareness, attitudes, and practices were used to measure them as binominal dependent variables. The logistic regression analysis model was applied to calculate the association between independent variables and dependent variables (KAP scores) of the community regarding rabies [4,16]. A 95% confidence interval (CI) of the adjusted odds ratio (AOR) and p-values was used to describe statistical significance associations. The association of KAP is judged as significant when the p-value is less than 0.05.

## Results

### Socio-demographic characteristics of respondents

A total of 140 community members were interviewed during the study period and all responded to the questionnaire. The majority of the respondents 83 (59.3%) were from rural, while that of urban was 57 (40.7%). Most of the respondent’s ages ranged from 18–30 years, which was 67 (47.9%), and of 31–45 years were 41 (29.3%). Similarly, 113 (80.7%) of respondents were males, 82 (58.6%) were married, 18 (12.9%) were unable to read/write, and 65 (46.4%) attended primary education. Moreover, 61 (44.8%) of respondents knew someone who had been exposed to rabies (Table 1).

**Table 1:** The study participant’s sociodemographic characteristics.

Variable	Category	Frequency	Percentage
**Residence**	Rural	83	59.3
	Urban	57	40.7
**Age**	18–30	67	47.9
	31–45	41	29.3
	> 45	32	22.9
**Sex**	Female	27	19.3
	Male	113	80.7
**Marital Status**	Single	48	34.3
	Married	82	58.6
	Divorced	10	7.1
**Educational status**	Unable to read/write	18	12.9
	Primary school	65	46.4
	Secondary school	44	31.4
	Diploma and above	13	9.3
**Religion**	Muslim	121	86.4
	Christian	19	13.6
**Occupation**	Farmer	69	49.3
	Daily laborer	11	7.9
	Student	15	10.7
	Merchant	35	25
	Civil servant	10	7.1
**Dog ownership**	Yes	54	38.6
	No	86	61.4
**Knowing someone exposed to the rabies virus**	Yes	60	44.8
	No	74	55.2

### Knowledge, attitude, and practice regarding Rabies among participant

The majority of respondents 134 (95.7%) had heard about rabies. A large number of the respondents 85 (63.4%) reported that they heard of the disease from non-mass media whereas 42 (31.3%) were from mixed sources. However, only 42 (31.3%) of the respondents knew the causative agent of rabies. Moreover, regarding the seasonal distribution of rabies, 65 (48.5%) knew that the disease occurs in summer in humans and animals, and 113 (84.3%) answered dogs and wild canines as a source of rabies. Regarding transmission, 130 (97%) of respondents admitted that dog bites are the main means of transmission in humans and animals and 119 (88.8%) knew that rabies can be prevented by regular dog vaccination. From a multiple-choice question asked about signs and symptoms of the disease in humans, about 105 (78.4%) of respondents believed a puppy sound heard from the abdomen of patients. Concerning signs and symptoms of rabies in animals, 110 (82.1%) knew the change in behavior as a main sign (Table 2).

**Table 2:** Knowledge, attitudes, and practices regarding rabies among participants.

Knowledge				Attitudes				Practice			
Variable	Category	Frequency	Percentage	Variable	Category	Frequency	Percentage	Variable	Category	Frequency	Percentage
**Have you ever heard of rabies?**	Yes	134	95.7	**Post-exposure prophylaxis prevents rabies disease development**	Yes	120	89.6	**When would you present to the hospital after a bite**	Immediately after being bitten	118	88.1
	No	6	4.3								
**From where have you heard of rabies**	Mass media	7	5.2								
	Non mass media	85	63.4								
	Mixed source	42	31.3						Between 2 to 14 days after being bitten	7	5.2
**Cause of the disease**	Virus	41	30.6		Do not know	14	10.4				
	Bacteria	23	17.2								
	Do not know	70	52.2						Would do nothing	9	6.7
**Season rabies is more common**	Summer	65	48.5	**Eating roasted meat of animals that died of rabies could be medicine for rabies**.	Yes	12	9.0	**How are wounds bitten by rabies-suspected animals managed?**	Herbal remedies	19	14.2
	Autumn	22	16.4								
	Winter	17	12.7								
	Spring	23	17.2								
	Do not know	7	5.2								
**Source of Rabies**	Dog	2	1.5		No	91	67.9		Post-exposure prophylaxis	113	84.3
	Dog and wild canine	113	84.3								
	Do not know	19	14.2								
**Animal species commonly affected by Rabies**	Dog only	3	2.2								
	Dog and Human	8	6.0		Do not know	31	23.1		Holy water	2	1.5
	Human and other domestic animals (Bovine, Equine, Caprine, Ovine)	117	87.3								
	Do not know	6	4.5								
**Is rabies transmitted from animal to human**	Yes	126	94.0	**Free-roaming or stray dogs are important in rabies transmission between dogs and between dogs and other animals/humans**.	Yes	123	91.8	**What are your practices for a rabid animal?**	Tie	13	9.7
	No	1	0.7								
	Do not know	7	5.2								
How is rabies transmitted to humans?	Bite by any rabid animal	130	97.0								
	Contact with saliva on an open wound	105	78.4		No	4	3.0		Killed by community	115	85.8
	Rabid animal respiration	48	35.8								
	Raw meat and milk	60	44.8		Do not know	7	5.2		Do not know	6	4.5
	Do not know	8	6.0								
**Preventable in animals, humans, or both by dog vaccination**	Yes	119	88.8	**May the exposure to rabies virus go unrecognized without showing any sign**	Yes	49	36.6	**If killed what action is taken with the carcass**	Throw it away	2	1.5
	No	4	3.0		No	48	35.8				
	Do not know	11	8.2		Do not know	37	27.6				
**Signs and symptoms of rabies in humans**	Paralysis	2	1.5	**Rabies is curable after onset of symptom**	Yes	68	50.7				
	Stop eating and drinking	25	18.6								
	Hypersalivation	52	38.8						Burn	5	3.7
	Hydrophobia	27	20.1								
	Puppy sound in the abdomen	105	78.4		No	55	41.0		Bury	108	80.6
	Do not know	19	14.2								
**Signs and symptoms of rabies in animals**	Salivation	36	26.7								
	Stop eating and drinking	9	6.7		Do not know	11	8.2		Do not know	19	14.2
	Change in behavior	110	82.1								
	Do not know	26	19.4								
**Is rabies virus fatal in nature?**	Yes	124	92.5	**Eliminating stray or confining dogs helps prevent rabies in human**	Yes	128	95.5	**Are you vaccinating your dogs against rabies once a year or not?**	Yes	5	3.7
	No	3	2.2								
	Do not know	7	5.2								
**The incubation period of the rabies**	Immediate	3	2.2								
	<40 days	24	17.9								
	>40 days	90	67.2		Do not know	6	4.5		No	129	96.2
	Do not know	17	12.7								

About 120 (89.6%) of the respondents answered that post-exposure prophylaxis can prevent rabies development. Of the total participants, 91 (67.9%) disagreed that eating roasted meat of animals that died of rabies could cure the disease. Moreover, 123 (91.8%) of the respondents agreed with the importance of free-roaming or stray dogs in rabies transmission to humans; 95.5% of the respondents agreed that eliminating stray dogs or confining free-roaming dogs helps to prevent rabies (Table 2).

**Fig 3:**
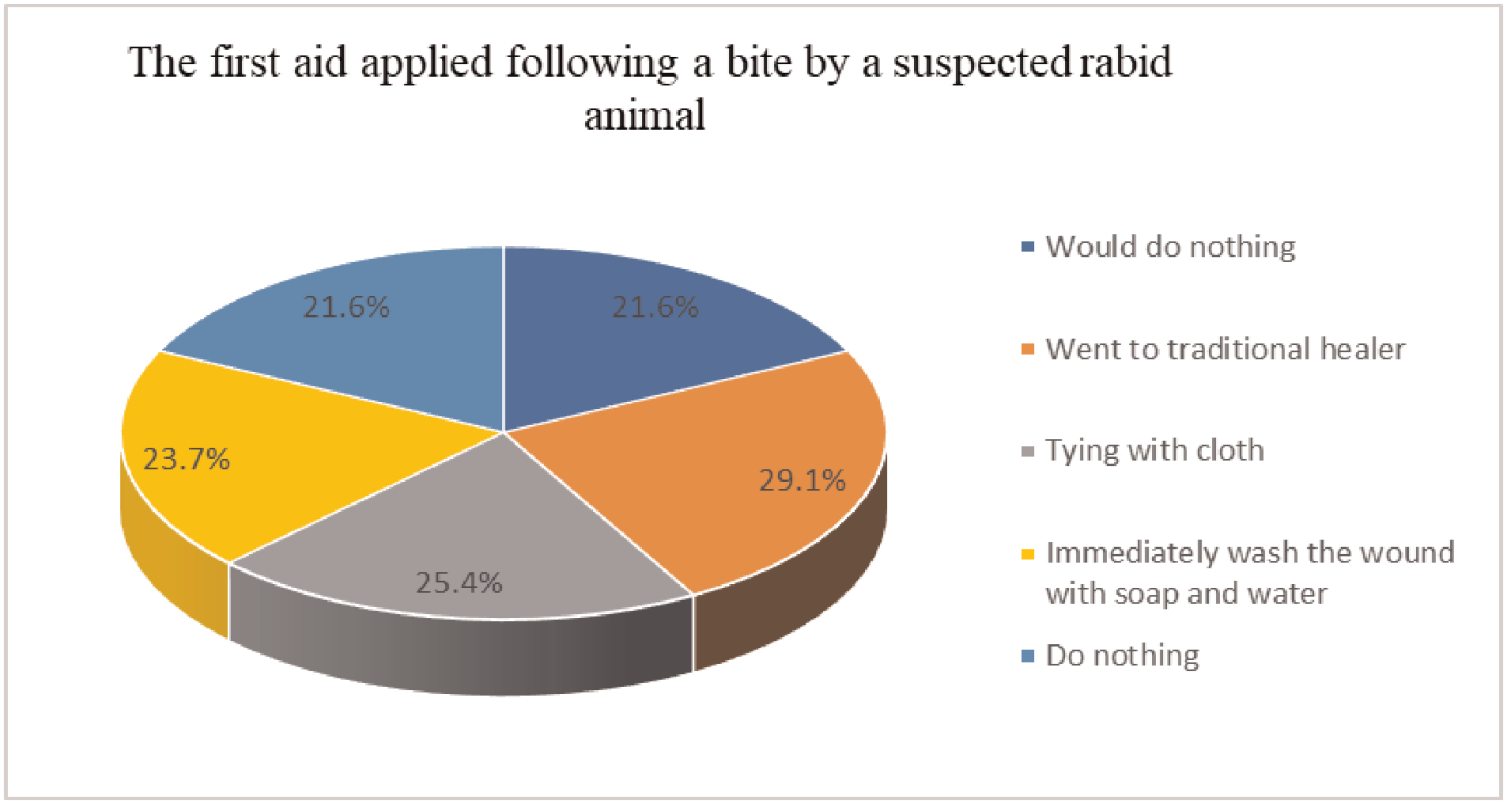
Practices of respondents following a suspected dog bite.

For first aid action taken for dog bites, about 39 (29.1%) went to traditional healers, and 34 (25.4%) tied the bitten area with cloth (Fig 3). Similarly, the result showed that the majority of the respondents 118 (88.8%) would present to health care immediately after being bitten by a suspected dog bite. About 113 (84.3%) of participants manage rabies-suspected bites with post-exposure and 19 (14.2%) would use herbal remedies. When asked about actions to be taken for the rabid animal, most respondents 115 (85.8%) reported that they would kill the animal, and 13 (9.7%) responded that they would tie the animal. Most respondents 108 (80.6%) reported that they would bury, whereas a considerable number of respondents 19 (14.2%) stated they did not know what would be done with the carcass. The vast majority of the respondents 49 (90.7%) would not vaccinate against the rabies virus their dogs regularly (Table 2).

### Community KAP level towards rabies in Gomma district

**Fig 4:**
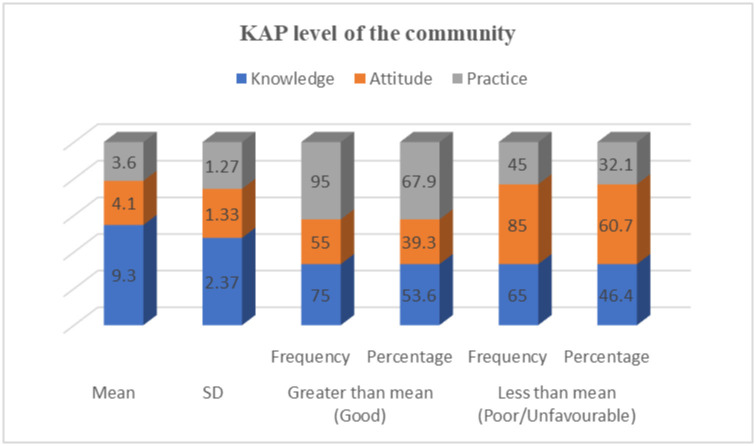
Mean, standard deviation, and frequency of community KAP levels regarding rabies.

The study showed that 75 (53.6%) of the participants involved in this study were found to have good knowledge about rabies and 55 (39.3%) of participants had favorable attitudes. In addition, 95 (67.9%) of respondents were found to have good practices (Fig 4).

### Factors associated with KAP regarding rabies

Variables including residence, educational level, religion, and knowing someone who had been exposed to rabies showed a significant knowledge score association at a *p*-value of 0.25 and incorporated in multivariable mixed effect logistic regression. The multivariable analysis result revealed the religion of the respondents was significantly associated with a higher good knowledge score of the respondents in which Christians had a higher knowledge score of 14 (73.7%) than Muslims 62 (51.2%). Christians were seven times more likely to have a good knowledge score compared to Muslims (AOR = 6.876, CI = 1.750–27.016, *p* = 0.006). A good knowledge score was also significantly associated with knowing someone who had been exposed to rabies. Respondents who knew someone’s exposure had a higher knowledge score of 45 (73.8%) than those who did not 31 (39.3%). Thus, those who knew had six times more good knowledge than those who did not (AOR = 6.208, CI = 2.750–14.012, *p* = 0.000) (Table 3).

**Table 3:** Multivariable logistic regression analysis of factors associated with KAP regarding rabies.

Variables	Category	Knowledge		AOR (95% CI of AOR)	*p*-values
		Good (%)	Poor (%)		
**Residence**	Rural	40 (48.2%)	43 (51.8%)	-	0.183
	Urban	36 (63.2%)	21 (36.8%)	1.717 (0.798, 3.915)	
**Education**	Unable to read/write	8 (44.4%)	10 (55.6%)	-	0.242
	Primary school	30 (46.2%)	35 (53.8%)	2.954 (0.718, 12.409)	
	Secondary school	29 (65.9%)	15 (34.1%)	4.313 (1.051, 17.977)	
	Diploma and above	9 (69.2%)	4 (30.8%)	4.193 (0.740, 23.500)	
**Religion**	Muslim	62 (51.2%)	59 (48.8%)	-	0.004
	Christians	14 (73.7%)	5 (26.3%)	6.826 (1.756, 27.116)	
**Knowing someone who had been exposed to rabies**	Yes	45 (73.8%)	16 (26.2%)	6.248 (2.790, 14.032)	<0.001
	No	31 (39.3%)	48 (60.7%)	-	
		**Attitude**			
		**Favorable (%)**	**Unfavorable (%)**		
**Religion**	Muslim	53 (43.8%)	68 (56.2%)	5.598 (1.189, 25.393)	0.026
	Christians	2 (10.5%)	17 (89.5%)	-	
**Knowing someone who had exposed been to rabies**	Yes	32 (52.5%)	29 (47.5%)	2.369 (1.167, 4.849)	0.019
	No	23 (29.1%)	56 (70.9%)	-	
		**Practice**			
		**Good (%)**	**Poor (%)**		
**Educational status**	Unable to read/write	17 (65.4%)	9 (34.6%)	**-**	0.286
	Primary school	19 (73.1%)	7 (26.9%)	1.930 (0.231, 5.301)	
	Secondary school	33 (80.5%)	8 (19.5%)	1.613 (0.872, 6.131)	
	Diploma and above	41 (87.2%)	6 (12.8%)	1.543 (0.027, 4.989)	
**Residence**	Rural	61 (73.5%)	22 (26.5%)	1.963 (0.332, 5.932)	0.115
	Urban	34 (59.6%)	23 (40.4%)	-	
**Age**	18–30	40 (59.6%)	27 (40.3%)	-	0.408
	31–45	31 (75.6%)	10 (24.4%)	1.681 (0.607, 4.329)	
	> 45	24 (75%)	8 (25%)	1.749 (0.675, 4.809)	
**Dog ownership**	No	63 (73.3%)	23 (26.7%)	1.491 (0.629, 3.333)	0.312
	Yes	32 (59.3%)	22 (40.7%)	-	
**Knowing someone who had been exposed to rabies**	No	47 (59.5%)	32 (40.5%)	-	0.259
	Yes	48 (78.7%)	13 (21.3%)	1.651 (0.670, 3.949)	
**Knowledge score**	Poor knowledge	40 (62.5%)	24 (37.5%)	-	0.420
	Good knowledge	55 (72.4%)	21 (27.6%)	1.394 (0.617, 3.128)	
**Attitude score**	Unfavorable attitude	49 (57.6%)	36 (42.4%)	-	0.005
	Favorable attitude	46 (83.6%)	9 (16.4%)	3.267 (1.391, 7.730)	

AOR: Adjusted Odds Ratio; %: Percentage

In attitude score, religion and knowing someone who had been exposed to rabies were the only two candidate variables in the multivariable mixed-effect logistic regression model. Both variables remain significant in multivariable logistic regression. Muslims were found to have six times more favorable attitudes than Christians (AOR = 5.518, CI = 1.199–25.391, *p* = 0.028). Those who knew someone’s exposure to rabies were found to have twice more favorable attitudes than those who didn’t (AOR = 2.367, CI = 1.157–4.839, *p* = 0.018) (Table 3).

In rabies-related practice of respondents, candidate variables entered into multivariable logistic regression were residence, age, dog ownership, knowing someone who had been exposed to rabies, knowledge score, and attitude score. The multivariable mixed effect logistic regression model of rabies-related practice of respondents showed attitudes towards rabies were the only significant predictor of practice. Those who had favorable attitudes towards rabies had three times more good practices than those who had unfavorable attitudes (OR = 3.267, CI = 1.391–7.730, *p* = 0.005) (Table 3).

## Discussion

Rabies virus is a generalist pathogen in nature as it has the potential to infect a wide range of animals and cause large host death. Rabies in Ethiopia is a neglected zoonotic disease but a serious public health burden, especially in locations where stray dogs are ineffectively managed [15].

The current study revealed that rabies is a commonly known human and animal disease in the study area. Of the 140 respondents, 95.7% had heard about rabies. This implies that the majority of the respondents in the study area are aware of the rabies impact on the public and animals and its commonness. This is similar to a study by Tiwari *et al*. [18] in the Panchkula district of north India who reported that 96% of respondents were familiar with general information on rabies. The finding is higher than a report by Ahmed *et al*. [25] in and around Adigrat town, Tigray regional state of Ethiopia, Ichhpujani *et al*. [26] in India, and Ali *et al*. [27] in Addis Ababa, Ethiopia who reported 67.5%, 68.7%, and 83%, respectively. The result is lower than the previous reports in Ethiopia by Yalemebrat *et al*. [28] in the Debark district, North Gondar, and Abdela *et al*. [29] in the Dedo district of Jimma zone who reported 100% awareness about rabies. This discrepancy may result from differences in health education and sources of information imparted on the knowledge of rabies infections between study areas [16,19,20].

The majority of respondents, 63.4% had heard about rabies from non-mass media including family, community members, friends, and elders. The reason may be due to rabies is a neglected tropical disease and no or few media programs deliver a message about the rabies virus to the public. The study showed that about 52.2% of respondents did not know what the cause of rabies was. This is similar to the study conducted by Kabeta *et al*. [30] in Jimma town who reported that 52.3% of respondents did not know the cause of the disease. The reason could be rabies-related lessons given only to human and animal health students at higher levels and there may be low awareness creation programs about the disease for the community at large. Furthermore, such information tended to be cursory, failing to provide the public with an adequate level of awareness about rabies. Birasa *et al*. [20] and Matibag *et al*. [19] found that the highest percentages of respondents received about rabies from several sources (health professionals, radio/television). The disparity could be attributed to variations in the community’s awareness level about the disease.

In the current study, regarding the seasonal distribution of rabies, about 48.5% answered that the disease occurs in summer more commonly. This agrees with a report of Shite *et al*. [31]. This may be due to the movement of canines in search of food, dogs released to crop farms to keep from wild animals, which could expose domestic dogs to wild canines, and the approach of the mating season that triggers their movement. Similarly, 87.3% of respondents knew that rabies could affect humans and other domestic animals. This is inconsistent with a study by Abdela *et al*. [29] in the Dedo district of Jimma zone, Ethiopia, Guadu *et al*. [13] from Bahir Dar town, Ethiopia, and Ahmed *et al*. [25] in and around Adigrat town, Tigray regional state Ethiopia, who reported 57%, 21.4%, and 67.7%, respectively. This could be due to differences in the awareness of the disease’s nature. Also, the variation could be due to the different socio-cultural settings of communities. This is similar to Klingen *et al*. [32] that bites of rabid animals let the virus inoculate into muscle and subcutaneous tissues with their saliva through the skin.

About 90.7% of respondents believe that rabies is preventable by regularly vaccinating dogs. This is in line with Hampson *et al*. [33] and Hildegund [34]. They stated successive annual mass dog vaccination campaigns that achieve 70% vaccination coverage bring rabies under control in reservoir populations. However, contrary to the study conducted in Nekemte town by Tolessa and Mengistu [35]. The variation may have resulted from the gap in the opportunity of respondents to obtain knowledge about rabies from different sources through exposure to meetings, idea exchange with the community, and at the workplace due to the nature of their work. Furthermore, this may be a result of a difference in awareness of the importance of vaccinating pets due to insufficient vaccine education.

Regarding the incubation period of rabies, greater than 40 days was mentioned by 67.2% of respondents. This agrees with Singh *et al*. [36] who stated incubation period or eclipse phase is highly variable from 2 weeks to 6 years (average: 2 to 3 months) according to the concentration of the virus, inoculation site, and density of innervation. Most of the respondents had misconceptions about the signs and symptoms of rabies in humans. In multiple questions asked the majority of respondents, 78.4% answered puppy sounds are heard in the abdomen. This may be the traditional healer in the area who provided the information. In contrast, despite having some knowledge of rabies, the majority of participants had very limited knowledge of rabies signs and symptoms, and less than 14.2% did not know these symptoms, in contrast to another study conducted in Gujarat, India, where most participants were able to identify the typical clinical signs of rabies in humans [37]. This could be due to discrepancies in research areas and community awareness levels about rabies.

The 89.6% of the study participants had a favorable attitude toward post-exposure prophylaxis and 14.3% did not know whether it prevents disease development or not. This variation of attitude between respondents could be a lack of information about the vaccine. This is supported by Khan *et al*. [16], who reported the KAP regarding rabies endemicity among community members in Pakistan, where 85.2% of respondents had a positive attitude toward receiving anti-rabies vaccinations. Regarding the common belief that roasted meat of an animal that died of rabies could be a medicine, about 67.9% disagreed with it. This contradicts the report of Dabuma *et al*. [12], who reported that most respondents believe that if an animal that has died of rabies is properly slaughtered and the carcass is burned and inhaled, it serves as one mechanism to cure animals infected with the disease. This implies the old idea is outdated in the community. Similarly, 91.8% of the respondents 91.8% agreed on the importance of free-roaming and stray dogs in the transmission of rabies. About 99.5% of respondents believed eliminating stray dogs and confining dogs helps to prevent rabies. This is inconsistent with the study by Abdela *et al*. [29] in which 68.8% of participants believed that rabies could not prevented by eliminating stray or confining dogs.

Only 36.6% of participants believed the disease might go unrecognized without showing any clinical signs for a long time. This finding contradicts the findings of Guadu *et al*. [13], who indicated that around 76.8% of respondents were aware of frequent clinical indications of rabies in animals in Bahir Dar Town and elsewhere [38]. This could let the community not seek health care services and make them believe one is free of the disease once the expected time of clinical signs passed. The majority of respondents 50.7% believed that rabies is curable after the onset of clinical signs. This belief may come from communities’ experience of recovering from much more severe and acute disease that shows apparent clinical signs. Rabies is 100% fatal once warning signs have appeared and the treatment is generally supportive [39].

In the current study, only 23.7% of participants immediately washed the wound with soap and water as a first aid following a suspected dog bite. In contrast, in a study conducted by Yalemebrat *et al*. [28] in the Debark district, about 76.5% of the respondents preferred washing wounds created by a suspected dog bite with soap and water. Similarly, Khan *et al*. [16] reported that 3.2% of victims would wash the wound with water and soap as first aid. This may be due to extension training variation and additionally, this treatment is cheap, readily available, and feasible for all to apply readily. The World Health Organization (WHO) also recommends wound washing and vaccination immediately for at least fifteen minutes after contact with a suspected rabid animal which can prevent almost 100% of rabies deaths along with other prevention measures including post-exposure prophylaxis [39].

About 87.1% of participants would present to the hospital immediately after being bitten. This is supported by Khan *et al*. [16], and Sudarshan *et al*. [40], who found that the majority of respondents sought medical attention immediately following the exposure. This good practice may be due to the possibility of finding healthcare centers near the study area. About 84.3% of respondents would manage wounds of suspected dogs bitted with post-exposure prophylaxis. This is in line with WHO recommendations [39]. The majority of respondents would practice killing rabid animals in the community (85.8%) and burying (80.6%) them. This good practice was accumulated experience of the community in the study area and it is important to reduce the impact of rabid animals on public health.

Only 3.7% had been vaccinating their dog regularly. This is by far the smallest practice compared to other scholarly reports. Accordingly, 71.1% and 46% of participants vaccinate their dogs in a report by Ahmed *et al*. [25], in Ethiopia and by Sambo *et al*. [11] in Tanzania, respectively. The reason could be related to the lack of a rabies control program implemented in the study area until declared recently dog-mediated human rabies deaths by 2030.

In the current study, even though the respondents knew the importance (90.7%) of the vaccine in rabies prevention, a small number (3.7%) of them practiced vaccinating their dogs. These are attributed to the absence of the vaccination campaign in the study area thus far. Conversely, practicing the anti-rabies vaccine for their dogs would promote public health safety at large.

Regarding knowledge, attitude, and practice scores of the participants towards rabies, 53.6%, 39.3%, and 67.9% had good knowledge, favorable attitudes, and good practices, respectively. This finding is supported by Dabuma *et al*. [12], who found 53.4% of the KAP analysis to be good. However, the current findings are inconsistent with a previous report in Mekelle City, Ethiopia by Hagos *et al*. [41] who reported 56.1%, 56.2%, and 61.3% of participants had good knowledge, positive attitudes, and good practice towards rabies respectively. This difference could be related to variations in the level of awareness in the community, differences in the prevalence of exposure to the disease, and different sizes.

Multivariable logistic regression revealed that knowledge score was significantly associated with religion (*p* = 0.006); Christian participants were seven (AOR = 6.9; 95%CI = 1.750–27.016) times more likely to have good knowledge of rabies than Muslim participants. This is similar to Yusuf and Kassa [42] who reported a KAP score significant association of religion being higher in Christians (*p* = 0.0001) than Muslims in and around Jigjiga, Somali Region, Ethiopia. This might be happened due to the sample size difference. Respondents who know someone who had been exposed to rabies had a statistically significant difference (*p* = 0.000) and six (AOR = 6.2; 95%CI = 2.750–14.012) times more likely to have good knowledge of rabies than those who did not know. This could be related to memorizing what had happened, said at that moment of someone’s exposure, measures taken, and results found.

Moreover, the attitude score was significantly associated with religion (*p* = 0.028). Muslim participants were six (AOR = 5.5; 95%CI = 1.199–25.391) times more likely to have a favorable attitude towards rabies than Christian participants. This is inconsistent with a study conducted by Kabeta *et al*. [30] who reported an insignificant association between religion and attitude (*p* = 0.932) among households in Jimma town. This might relate to religious differences between the two religions about dogs, dog management, and variables included in the questionnaire. Participants who know someone who had been exposed to rabies also significantly associated with an attitude score (*p* = 0.018); being those who know someone exposed to rabies were twice (AOR = 2.4; 95%CI = 1.157–4.839) more likely to have a favorable attitude towards rabies than those who did not know. This agrees with the statement by Ellen [43] who stated that experience leads to a change in health behavior. The possible reason might be observation of someone’s exposure helps to improve attitude towards rabies.

In rabies-related practice, only attitude score was significantly associated with practice score (*p* = 0.008); being respondents who had favorable attitudes were more likely to have good practice towards rabies than those who had unfavorable attitudes. This is similar to the study of Ali *et al*. [27] who reported a strongly significant positive correlation between attitude and practice (*p* < 0.0001) among residents in Addis Ababa, Ethiopia. This is because a positive attitude modifies one’s practice. Practice towards the prevention of disease is improved by promoting attitude since attitude influences one’s behavior [44].

This study revealed that rabies remains a significant public health challenge in the studied community, particularly in areas with poor management of stray dogs. Despite high awareness of rabies among respondents, there are gaps in understanding its causes, symptoms, and prevention measures. Effective rabies mitigation requires comprehensive health education, emphasizing the importance of regular dog vaccinations and prompt post-exposure prophylaxis. Increased media coverage and community outreach programs are essential to improve public knowledge and attitudes. Moreover, enhancing collaboration between health professionals and the community can lead to better practices, ultimately reducing the incidence and impact of rabies in affected regions.

### Limitations of the study

The study’s limitation lies in not including respondents from various sectors like human, animal, and environmental health to compare their KAP levels due to time, financial, and manpower constraints. Additionally, the study relied solely on interview/questionnaire data without collecting clinical samples. The primary aim was to assess the community’s KAP level regarding rabies and provide insights for stakeholders to mitigate the disease’s impact in the study area using a small sample size with available resources.

## Conclusion

Rabies significantly impacts both animals and humans in the study area. The respondents exhibited limited knowledge and inconsistent attitudes and practices regarding rabies prevention and control. Those aware of the disease had good knowledge and attitudes but demonstrated poor practices. A significant knowledge gap was identified concerning the cause, clinical signs, and prevention of rabies. This lack of awareness could negatively affect public and animal health, with annual dog vaccinations not practiced in the area. Religion and exposure history significantly influenced the communities’ knowledge and attitude scores, while the attitude score was significantly associated with practice scores. Enhancing rabies vaccination coverage and canine rabies control is necessary to reduce the prevalence of this deadly zoonotic disease in the study area and Ethiopia at large. Accordingly, comprehensive public health campaigns are essential to achieve these goals. Therefore, to effectively control and manage rabies, it is crucial to raise awareness among community members; Stakeholders should collaborate to educate the community, establish a One Health committee to address rabies and other zoonotic diseases, and disseminate rabies-related information through local and national media; Additionally, extensive disease surveillance across the country is encouraged to measure the actual burden of rabies and implement effective control measures.

## Supporting information

S1 Table De-identified dataset was used for all analyses presented in this manuscript. (XLS)
